# Domestic Correspondence

**Published:** 1890-10

**Authors:** 


### Domestic Correspondence.

To the Editor:

Dear Sir,—As the readers of your journal have been informed of an "incident," said to have taken place at a meeting of the New York Odontological Society, in March, I find in looking it over that it is misrepresented. I cannot forego the opportunity of correcting it, for I am the person, "not a member," that related the case.

Matthew Arnold says, "He who assumes to criticise must at the time rise superior to the one he aims to criticise." When I am done with this I will leave the reader to decide who is criticised. I consider the whole statement of the writer simply one of special pleading. If the presentment had been made in the spirit I gave it, the impression would not have been so misleading. Much that I did say was left out, which would have contradicted what the writer said. Much that is given I did not say.

I prefaced my remarks by stating that I well knew the difficulty of relating such an incident, so as not to give a wrong impression, and avoid the suspicion of a personality. I presented it for the purpose, I said, of emphasizing the criticisms given by Dr. Shepard in his late paper, which were directed to the importance of a change for the better in the management of our College Infirmaries. I am too much in love with the best interests of our calling, and feel too kindly towards men that have done so much unthanked service in our schools, to think of injuring them in any way. I am not a superficial observer of what constitutes an intelligent care of the mouth as viewed from the advanced standard of to-day. I simply stated that this patient was an intelligent person; and had confided in a practitioner for many years the full care of her case, and that he was a much reputed teacher in one of our leading dental colleges.

I did not criticise the mechanical part of his service as exhibited by fillings, but did the unhealthy condition of the mouth, finding as I did so many dead pulps—and offensive ones, as they proved—and the advanced stage of the distressing disorder, "Riggs's disease," and, as the patient said, having never had her attention called to the fact. One of her molars was so loose I took it away with my fingers, and found the palatal root absorbed, and as sharp as a needle point, causing agonizing pains at the base of the brain. On the opposite side two molars were very loose, and with mal-occlusion and sorely painful.

I think I have said enough, and I will put it beside the article referred to, and let intelligence give the verdict.

The question is raised by the writer regarding the fairness of reporting such cases. As we are not yet a liberal profession, as shown in many quarters, and as we have much crude material to deal with, I think a little friction on a dry surface will serve as a counter-irritant and induce a better resolution, which will bring things ultimately into healthier conditions. Of course, this must be mixed with a good deal of fraternal spirit, certainly more than is evidenced by "X." The multitude of adjectives that "X" injected, such as "prominent," were not used by me. If "X" had, while he was about it, with so much solicitude for courtesy, continued his correspondence of a domestic character, and given another incident that occurred at the same meeting, by a member, he would have improved his article.

G. A. MILLS.

NEW YORK, May 9, 1890.

[The foregoing is admitted at the special request of Dr. Mills, who felt aggrieved at the allusions of "X" in "Domestic Correspondence."—ED.]

---

### POST-GRADUATE DENTAL ASSOCIATION.

THE annual meeting of the Post-Graduate Dental Association of the United States was held at Chicago, June 25, 1890, and the following gentlemen were elected officers: President, George H. Cushing, M.D., D.D.S., Chicago, Ill.; Vice-President, Dr. R. H. Cool, Oakland, Cal.; Secretary and Treasurer, Lewis S. Tenney, D.D.S., Chicago, Ill.

*Executive Committee.*—R. B. Tuller, D.D.S., Chicago, Ill.; Dr. J. M. Gallehugh, Chenoa, Ill.; Dr. G. W. Milton, Silvertown, Col.

This association is but a year old, but it starts out with good prospects of becoming a large and popular national organization, and has a grand work before it. Its object, aside from the same general one of most dental societies, is to particularly encourage and stimulate post-graduate studies and the establishment of facilities for the same in dental colleges. It also contemplates, when its membership will admit of it, establishing a systematic course of home study with benefits not unlike the Chautauqua Literary Society, perhaps, but the plan is not yet sufficiently developed to admit of outlining at this time.

While the name "Post-Graduate" would imply an association of graduates only, the broad view is adopted of extending the work among all legal practitioners who may desire to join and co-operate; but practitioners not graduates are not eligible to membership until they have passed a post-graduate or practitioner's course in some reputable and recognized dental college.

Members of the profession who desire to become members of the Post-Graduate Association should correspond with the Secretary, Dr. Lewis S. Tenney, 96 State Street, Chicago. The membership fee is $1. Annual dues, payable in advance, $1. Certificates of membership are issued when the member duly qualifies. Membership may be obtained through correspondence when evidence of eligibility is presented.

---

To the Editor:

The following are the names of the officers of the Minnesota State Dental Association, held July 9–11, 1890: President, M. G. Jenison, Minneapolis; Vice-Presidents, W. C. Merrill, Albert Lea; Secretary, L. D. Leonard, Minneapolis; Treasurer, H. M. Reid, Minneapolis; Chairman Executive Committee, E. F. Clark, Minneapolis (elected); Master Clinics, C. A. Van Duzee, St. Paul (appointed by President J. M. Welch, St. Paul); E. K. Clements, Faribault; T. E. Weeks, Minneapolis; Membership Committee, F. H. Brimmer, Minneapolis; L. W. Lyon, J. H. Martindale, L. C. Davenport, M. R. Metcalf.

Yours,

L. D. Leonard, *Secretary.*

